# Photodynamic Therapy and Multi-Modality Imaging of Up-Conversion Nanomaterial Doped with AuNPs

**DOI:** 10.3390/ijms23031227

**Published:** 2022-01-22

**Authors:** Wei Zhang, Yanli Lu, Yang Zang, Jinhui Han, Qingyun Xiong, Jinping Xiong

**Affiliations:** 1 Beijing Key Laboratory of Electrochemical Process and Technology of Materials, School of Materials Science and Engineering, Beijing University of Chemical Technology, Beijing 100029, China; 2019200387@mail.buct.edu.cn (W.Z.); 2019310036@mail.buct.edu.cn (Y.L.); 2019310030@mail.buct.edu.cn (Y.Z.); 2020200494@mail.buct.edu.cn (Q.X.); 2 State Key Laboratory of Organic-Inorganic Composites, School of Chemical Engineering, School of Materials Science and Engineering, Beijing University of Chemical Technology, Beijing 100029, China; 2021400008@buct.edu.cn; 3 College of Ecology and Resources Engineering, Wuyi University, Wuyishan 354300, China

**Keywords:** up-conversion, nanomaterials, photodynamic, multi-modality imaging

## Abstract

Two key concerns exist in contemporary cancer chemotherapy in clinic: limited therapeutic efficiency and substantial side effects in patients. In recent years, researchers have been investigating a revolutionary cancer treatment technique, and photodynamic therapy (PDT) has been proposed by many scholars. A drug for photodynamic cancer treatment was synthesized using the hydrothermal method, which has a high efficiency to release reactive oxygen species (ROS). It may also be utilized as a clear multi-modality bioimaging platform for photoacoustic imaging (PAI) due to its photothermal effect, computed tomography (CT), and magnetic resonance imaging (MRI). When compared to single-modality imaging, multi-modality imaging delivers far more thorough and precise details for cancer diagnosis. Furthermore, Au-doped up-conversion nanoparticles (UCNPs) have an exceptionally high luminous intensity. The Au-doped UCNPs, in particular, are non-toxic to tissues without laser at an 808 nm wavelength, endowing the as-prepared medications with outstanding therapeutic efficacy but exceptionally low side effects. These findings may encourage fresh effective imaging-guided approaches to meet the goal of photodynamic cancer therapy to be created.

## 1. Introduction

Interest in developing theranostic nanoplatforms with simultaneous diagnostic and therapeutic capacity has gradually increased in the nanomedicine field because it provides significant prospects in the treatment of major illnesses including cancer [1]. Imaging probes, as one of the most important components of the theranostic nanoplate-form, should be able to perform many levels of imaging at the same time, from the cell to the whole body, to offer comprehensive tumor characteristics for clinical diagnostics. However, single-modality imaging did not match the high diagnostic criteria since each imaging technique (optical imaging, CT, and MRI) has intrinsic flaws due to restricted resolution, sensitivity, or imaging depths.

To mitigate this problem, several imaging probes were combined into a single multi-modality imaging system, which contained some considerable restrictions such as sophisticated synthetic processes and heterogeneous nanostructures. As a result of their enhanced optical and magnetic properties, and also improved X-ray attenuation, lanthanide-doped upconverting nanoparticles (UCNPs) might be perfect for building multifunctional bio probes by doping with various rare earth ions without modifying other functions.

Many researchers have recently advocated that this system be employed in biological imaging since it provides considerable benefits in the treatment against major illnesses such as cancer. However, the typical challenge is that it has insufficient light intensity and is poisonous to biological cells; therefore, its structure and surface must be modified [2]. Many scholars have proposed doping Mo^3+^, Cu^2+^ [3,4], and other metal ions in the NaYF_4_:Yb^3+^/Er^3+^ unit cell to increase the luminous intensity [5,6], but the effect is not significant. Others have offered sliver doping [7], which has a large impact as well; however, sliver is poisonous to cells, may cause cell death without targeting, and cannot be employed in biology. Many scholars have proposed constructing core-shell structures such as NaYF_4_:Yb^3+^/Er^3+^@NaGdF_4_:Yb^3+^ and NaYF_4_:Yb^3+^/Er^3+^@NaNdF_4_:Yb^3+^/Tm^3+^@NaGdF_4_:Yb^3+^ [8,9,10]. Alternatively, use the reverse microemulsion method to construct a layer of silica or porous silica, such as NaYF_4_:Yb^3+^/Er^3+^@SiO_2_, or NaYF_4_:Yb^3+^/Er^3+^@NaGdF_4_:Yb^3+^@m-SiO_2_ [11,12,13,14]. Au nanoparticles (AuNPs) are currently the mainstream biomaterials in tumor diagnosis and treatment applications [15,16]. They are widely used in CT imaging and photoacoustic imaging due to their excellent imaging capabilities and photothermal effects [17]. However, in our research, their photothermal stability seems not to be very good [18]. Unfortunately, when these materials meet the biological requirements, they will inevitably reduce their luminous intensity, so that imaging cannot be performed to obtain a clear image [19,20]. Considering the high desire to develop UCNPs nanomaterials with highly effective imaging capability as well as high biocompatibility to prevent apoptosis or biological organ failure, UCNPs doped with Au nanoparticles (AuNPs) are an ideal candidate because they are easy to fabricate, have enhanced luminescence, and are easy to surface modify [7,21]. More notably, following illumination, the UCNPs are harmless to normal tissues but cytotoxic to malignancies. To the best of our knowledge, however, there appears to be a failure in the literature to yet create theranostic nanoplatforms integrating multi-modality bioimaging with light trigger chemotherapy.

## 2. Results and Discussion

The TEM images of gold nanoparticles (Figure S1) prepared using the hydrothermal method show that they are spherical and have an average diameter of 5 nm. TEM images show the morphology of Au-UCNPs (Figure S2). They are rod structures with a length of 50–100 nm and have narrow ends. The reason for this phenomenon is that during the nucleation and growth of the nanoparticles by the coprecipitation–hydrothermal method, the temperature controls its width and shape, and the time determines its length. When the temperature starts to drop, the two ends of the nanorod begin to shrink with the decrease in temperature, and finally show the phenomenon of narrowing at both ends. The successful doping of gold nanoparticles into UCNPs was proved by energy spectrum (Figure S3).

**Figure 1:**
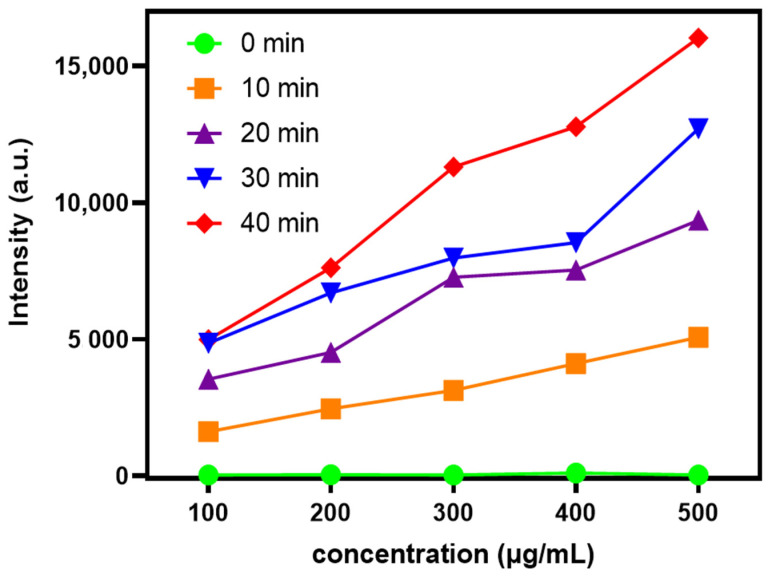
ROS generation by different concentration of Au-UCNPs-DSPE-PEG_2K_ was evaluated under near infrared irradiation at 808 nm for different time.

The content of ROS released by different concentrations of Au-UCNPs-DSPE-PEG_2K_ under near-infrared light with wavelength of 808 nm was measured by Singlet Oxygen Sensor Green (SOSG). As can be seen from Figure 1, Au-UCNPs-DSPE-PEG_2K_ hardly releases ROS without irradiation. The amount of ROS increased with the increase in Au-UCNPs-DSPE-PEG_2K_ concentration and time.

**Figure 2:**
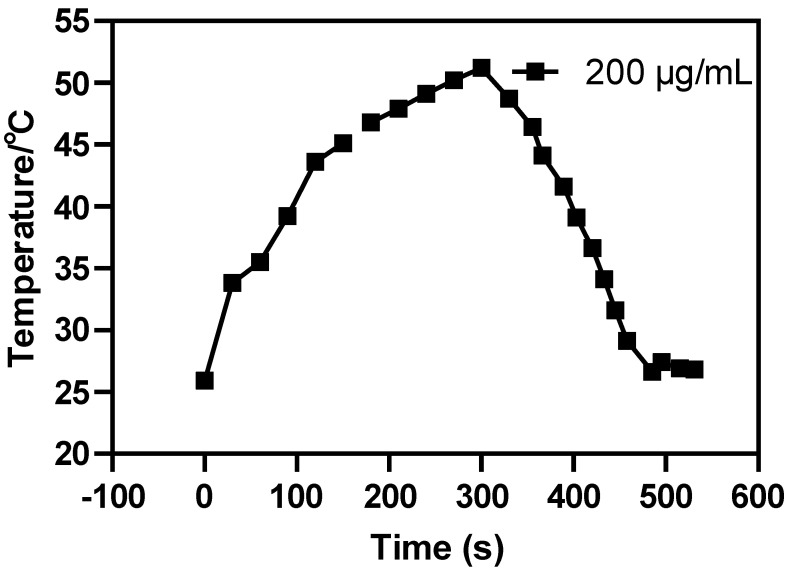
The 980 nm wavelength of near-infrared light excites the temperature rising-falling curve of 200 µg/mL of Au-UCNPs-DSPE-PEG_2K_.

Compared with the contrast ability of Au-UCNPs-DSPE-PEG_2K_, its particularity lies in its photothermal conversion efficiency (Figure 2). It is excited by near-infrared light at a 980 nm wavelength, it is observed by a thermal imager that it not only emits green fluorescence [21], but also emit heat. Combined with its biocompatibility, Au-UCNPs-DSPE-PEG_2K_ can be considered as a photothermal therapy reagent (Figures S4 and S5). The reason for this phenomenon is that gold will generate heat when irradiated by a 540 nm laser, while rare earth up-conversion nanomaterials excited by a 980 nm near-infrared light will emit 540 nm fluorescence. Secondly, the doping of silver nanoparticles will enhance the energy of a 540 nm wavelength. Finally, the energy of a 540 nm wavelength excites the silver nanoparticles, making the silver nanoparticles release heat. Incidentally, the carrier properties of a 540 nm laser, a 980 nm near-infrared light, and 540 nm fluorescence are the same, and the photothermal results also show that. Simultaneously, AuNPs enhance the luminescence intensity of UCNPs under the near-infrared light with the wavelength of 980 nm, so that UCNPs emit stronger light energy with the wavelength of 540 nm. This light energy further excites AuNPs, resulting in the heat emission of UCNPs doped with AuNPs [22].

**Figure 3:**
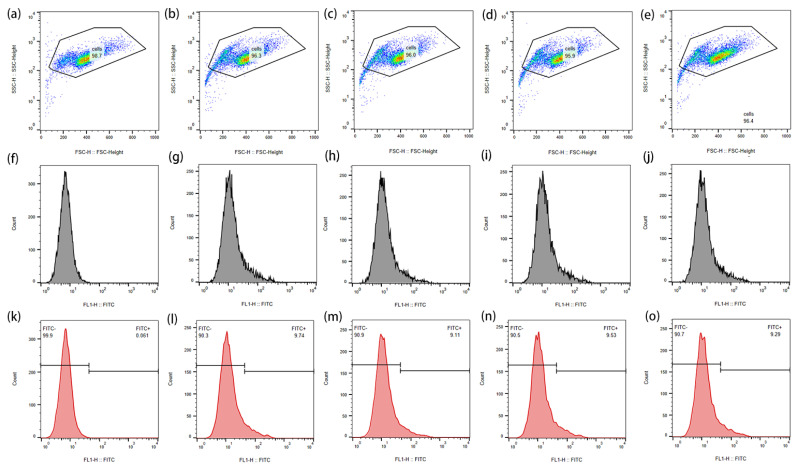
The effect of ROS produced by Au-UCNPs-DSPE-PEG_2K_ under laser on cells and the change in fluorescence intensity after DCFH-DA staining were measured by flow cytometry: (**a**): blank; (**b**–**e**): laser for 0 min, 10 min, 20 min, and 30 min, respectively, after adding Au-UCNPs-DSPE-PEG_2K_; (**f**–**j**): corresponding to the fluorescence intensity of (**a**–**e**,**k**–**o**): the negative and positive areas of (**a**–**e**).

In order to observe the ROS produced by Au-UCNPs-DSPE-PEG_2K_ and its effect on cells more intuitively, it was monitored by flow cytometry (Figure 3). ROS are molecules that contain hydroxyl radicals or peroxides with unpaired electrons. In healthy aerobic cells, ROS is naturally generated at a controlled rate as oxidation products of oxidative phosphorylation, oxidoreductase, or metal catalysis. However, it may be induced under some stress conditions, especially exposure to environmental oxidants and some drugs leading to release ROS. Excessive ROS may destroy cellular components including DNA, proteins, and lipids, and eventually lead to cell death. Cell permeability 2′,7′-dichlorodihydrofluorescein diacetate (DCFH-DA) is a widely used ROS indicator. The reduced non-fluorescein h2dcfda can be oxidized by intracellular ROS and converted into fluorescent 2′,7′-dichlorofluorescein (DCF). Therefore, Figure 3 labels intracellular ROS with DCFH-DA and detects the strength of DCF by flow cytometry.

**Figure 4:**
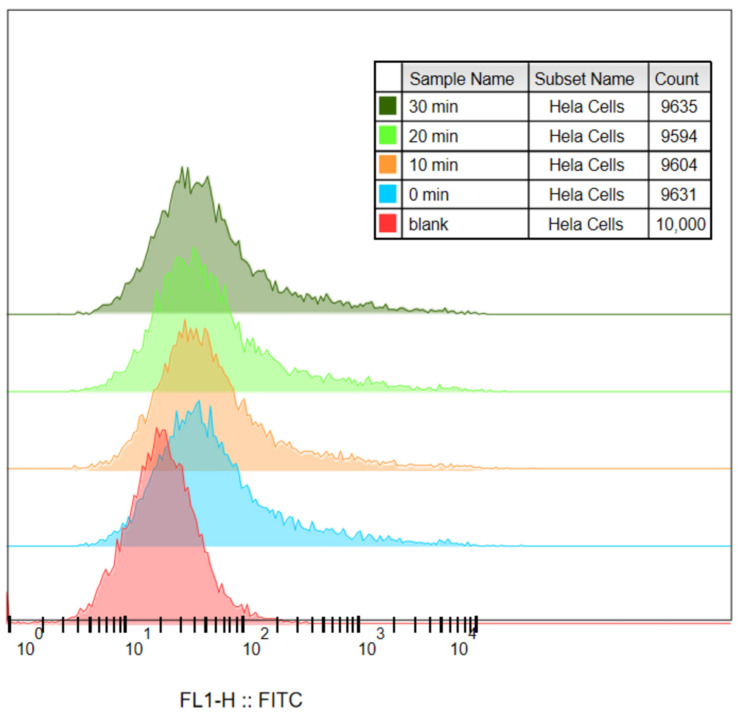
Histogram of Figure 3a–e.

It can be seen from Figure 3a–e that the cells initially gathered at one place. After adding Au-UCNPs-DSPE-PEG_2K_ and laser, there were two groups in the cell community, which indicates that under the laser, Au-UCNPs-DSPE-PEG_2K_ produced a large amount of ROS (Figure 1). A large amount of ROS destroyed the cells and turned the cells into fragments, resulting in two groups of cell communities, that is, one group was cell fragments. Figure 3f–j also confirmed this, which can be seen, with the addition of Au-UCNPs-DSPE-PEG_2K_ and laser, the fluorescein peak shifted to the right, that is (Figure 4), the fluorescence was enhanced. In other words, the amount of dye DCFH-DA converted into DCF by ROS increased.

**Figure 5:**
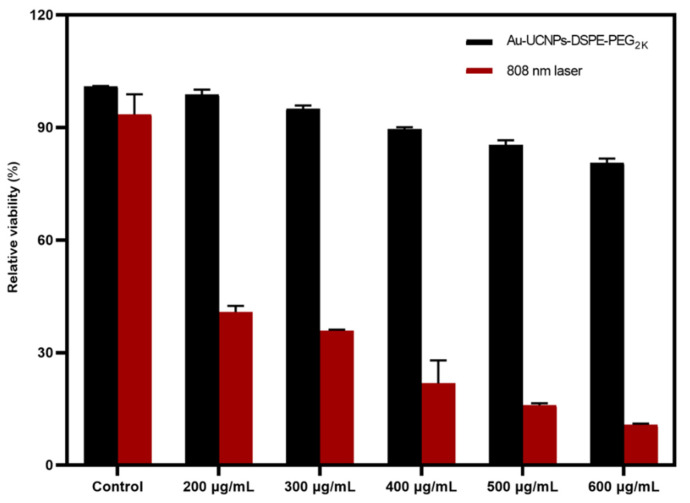
Cytotoxicity of different concentrations of Au-UCNPs-DSPE-PEG_2__K_ and in 808 nm laser.

The cytotoxicity of Au-UCNPs-DSPE-PEG_2K_ is tested by enzyme labeling instrument. HeLa cells are cultivated for 4 h after dispersing modified rare-earth nanomaterials in normal saline to prepare various quantities, and their activity is assessed (Figure 5). The cell survival rate is greater than 89% at the concentration of Au-UCNPs-DSPE-PEG_2K_ is less than 400 µg/mL. Particularly, at 200 µg/mL, the cell survival rate is greater than 99%. According to Figure 2, a 200 µg/mL concentration of rare-earth nanoparticles not only has appropriate safety but also has a high luminous intensity. Even when the rare-earth ion concentration is as high as 500 or 600 µg/mL, cell survival remains greater than 80%. Moreover, under the irradiation of near-infrared light with the wavelength of 808 nm, ROS was produced, which caused the apoptosis of cells (Figure S6).

**Figure 6:**
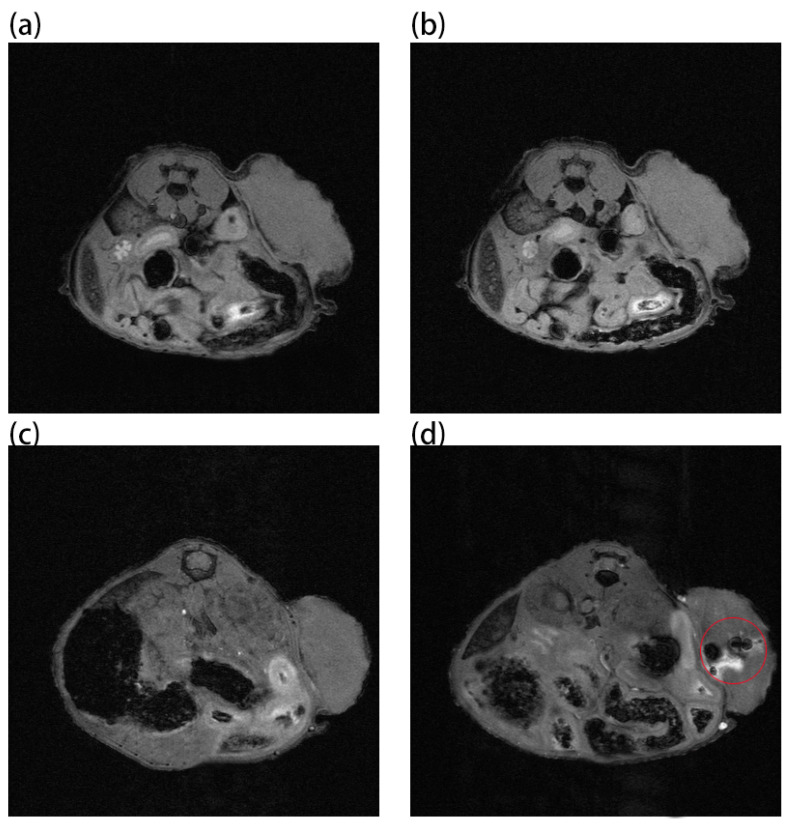
MR images before and after intratumor injection of Au and Au-UCNPs-DSPE-PEG_2__K_ in Balb/c mice, (**a**,**b**) are before and after injection of Au, (**c**,**d**) are before and after injection of Au-UCNPs-DSPE-PEG_2__K_, and MR value shown in red circle in (**d**).

Au and Au-DSPE-PEG_2K_ nanoparticles are injected intravenously into Balb/c mice (Figure 6). As can be seen from Figure 6a,b, there is no difference in MRI images before and after Au nanoparticles injection, demonstrating that AuNPs have no MRI imaging capabilities due to their lack of X-ray attenuation. After injection of Au-UCNPs nanoparticles, obvious MRI signals appear at the tumor location (in the red circle in Figure 6d), which is due to the X-ray attenuation characteristics of UCNPs (Figure S7).

**Figure 7:**
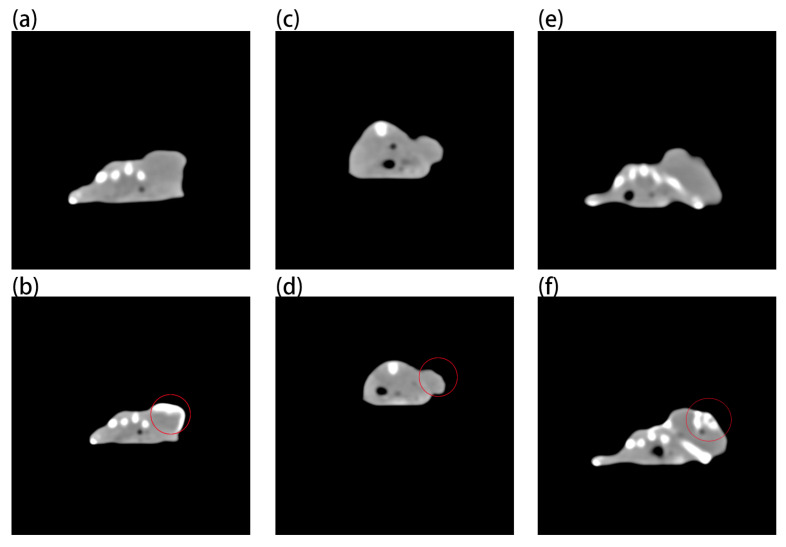
Micro-CT images before and after intratumor injection of Au and Au-UCNPs-DSPE-PEG_2__K_ in Balb/c mice, (**a**,**b**) are before and after injection of high concentration (50 mg/mL) of Au, (**c**,**d**) are before and after injection of low concentration (200 µg/mL) of Au, (**e**,**f**) are before and after injection of 200 µg/mL of Au-UCNPs-DSPE-PEG_2__K_, and CT value shown in red circle in (**b,d,f**).

Figure 7 shows that, although AuNPs have imaging ability (Figure 7b), it needs a very high concentration, while Au-UCNPs needs a low concentration (Figure S8), which is why very obvious CT signal is detected (Figure 7f). AuNPs and Au-UCNPs are at the same concentration, and Au cannot observe CT signal (Figure 7d).

**Figure 8:**
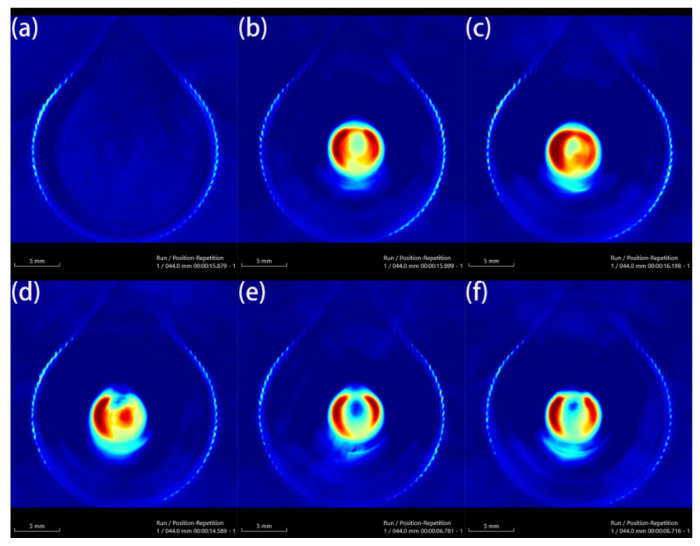
Photoacoustic imaging (PAI) of different concentrations of Au-UCNPs-DSPE-PEG_2K_, (**a**): 0 µg/mL, (**b**): 100 µg/mL, (**c**): 200 µg/mL, (**d**): 300µg/mL, (**e**): 400 µg/mL, (**f**):500 µg/mL.

**Figure 9:**
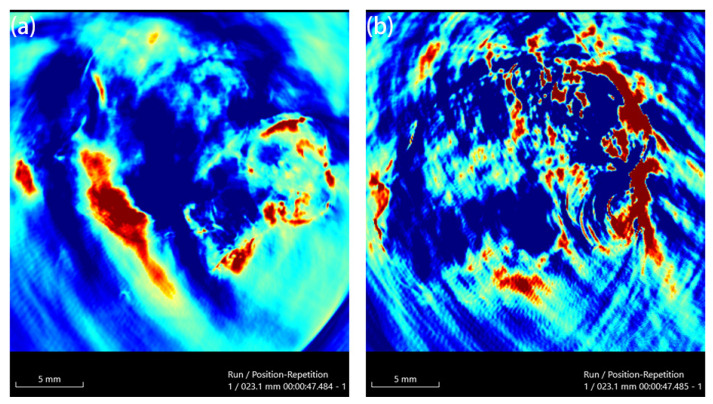
PAI of before (**a**) and after (**b**) injection 200 µg/mL of Au-UCNPs-DSPE-PEG_2K_ in Balb/c mice.

Au-UCNPs-DSPE-PEG_2K_ has excellent photoacoustic properties because of its excellent photothermal effect, which are characterized by photoacoustic imaging (Figure 8), and strong photoacoustic signals can be observed. Figure 9 shows that when injection the concentration of Au-UCNPs-DSPE-PEG_2K_ is 200 µg/mL, PA value is very obvious.

## 3. Materials and Methods

### 3.1. Materials

The Au nanoparticles and Au-UCNPs-DSPE-PEG_2K_ were synthesized in Beijing Key Laboratory of Electrochemical Process and Technology of Materials, Beijing University of Chemical Technology, Beijing, China [23]. Hela cells and BALB/c female white mice with SPF grade come from Beijing Laboratory of Biomedical Materials, Beijing University of Chemical Technology, Beijing, China. The cell counting kit 8 (CCK-8) assay kit was acquired from BOVOGEN Shanghai, China. All chemicals were utilized in as-received condition, without further refinement.

### 3.2. Characterization of Materials

A spectrum analyzer (ANDO AQ6317, Yokohama, Japan) was used to obtain the up-conversion luminescence spectra. The specimen was positioned in a 1.0 cm path length support and excited by utilizing a 980 nm CW semiconductor diode laser (Pmax 800 mW, 1000 mA). The up-conversion luminescence spectrum was acquired through the spectrophotometer having a multimode fiber with a core diameter of 0.6 mm. The top of the fiber was ~2 mm away from the specimen. Thermal imager (FOCUS 280DS) was used to characterize the photoacoustic properties of photographic materials. HORIBA laser and power density meter are used to characterize photothermal properties.

### 3.3. CCK-8 Assay for Cytotoxicity

HeLa cells were cultured in the logarithmic growth phase, and the culture medium was sucked out from the flask. The cells were then washed with PBS, and digested with the help of 0.25% trypsin. Then the trypsin was removed, and the cells were blown with DMEM media containing 10% fetal bovine serum before being shifted to the sample tank and blown well. Following that, 100 µL cells were introduced onto a 96-well plate (1 × 10^4^ cells/well) and cultured for 24 h at 37 °C in a constant temperature incubator (5% CO_2_). The cells were cultured in an incubator at 37 °C with 5% CO_2_ for 1.5 h at concentrations of 200, 300, 400, 500, and 600 µg/mL of Au-UCNPs-DSPE-PEG_2K_, respectively. The culture media was blotted out, PBS was washed twice, and the culture medium in the 96-well plates was replaced with 100 µL of fresh DMEM containing 10% fetal bovine serum, followed by 10 µL of CCK-8 solution in each well. After 2 h of incubation, the absorbance of each well at 450 nm was measured with a microplate reader.

### 3.4. SOSG Assay for ROS

Firstly, 100 µg SOSG was dissolved in 6600 µL of oxygen-free methanol solution and prepared as a mother liquor with a concentration of 15 µg/mL, and then stored away from light for later use. Then, a sample solution (200 µg/mL) was prepared, 100 µL was taken and mixed in a 96-well plate with 50 µL the SOSG mother liquor, and then irradiated with near-infrared light with a wavelength of 980 nm (irradiance 0.5 W/cm^2^) for 0, 10, 20, 30, and 40 min, each concentration was set to three repeated values and finally passed the spectrometer, which measured the fluorescence intensity of SOSG at 525 nm, and also detected other concentrations (300, 400, 500, and 600µg/mL) in the same way.

### 3.5. Establishment of Animal Tumor Model

A BALB/c female white mouse with SPF grade weighing 18 g was depilated, and log phase Hela cells were subcutaneously injected into the mice’s upper right hind leg to create a mouse Hela subcutaneous tumor development model.

### 3.6. MRI, PAI, and CT Imaging of Mice

To acquire preimages, the mice were anesthetized with isoflurane during the procedure, then placed in an animal MRI scanner (NM42-040H-I) with a magnetic field strength of 1 T, and a tomographic scan was executed of the tumor location on mice whose tumor developed to 100 mm^3^. Then, 200 µg/mL of Au-UCNPs-DSPE-PEG_2K_ solution was injected, and images were collected again. Similarly, the mouse was placed on a SPECT/CT (tube current: 615 μA, tube voltage: 55 kV) animal bed, a preimage acquisition of full-angle CT imaging in precise mode was performed, and then the tomographic image of the tumor site was acquired again after the injection of 200 µg/mL of Au-UCNPs-DSPE-PEG_2K_ solution. Using the same method, the images of mouse tumors before and after injection (200 µg/mL of Au-UCNPs-DSPE-PEG_2K_ solution) were obtained in the small animal photoacoustic imaging system (Nexus 128).

## 4. Conclusions

An Au-UCNPs-DSPE-PEG_2K_ multi-modality bioimaging device was ultimately developed and may be utilized for photodynamic treatment. The combined PA imaging with CT and MRI experiments show that Au-UCNPs-DSPE-PEG_2K_ may be used as contrast mediators for tri-modal imaging for both in vitro and in vivo testing, giving complete details for tumor diagnosis. Particularly, Au-UCNPs-DSPE-PEG_2K_ has a good release of ROS to destroy tumor cells to achieve the purpose of tumor treatment. These nanomaterials have exhibited low cytotoxicity, indicating their high biocompatibility for organisms. All these promising findings make Au-UCNPs-DSPE-PEG_2K_ nanocomposites an auspicious candidate for cancer theranostics, and it has encouraged us to develop the integration of diagnosis and treatment of tumors.

## Data Availability

No new data were created or analyzed in this study. Data sharing is not applicable to this article.

## Supplementary Materials

The following supporting information can be downloaded at: https://www.mdpi.com/article/10.3390/ijms23031227/s1.
