# Supplementary material for: Photodynamic Therapy and Multi-Modality Imaging of Up-Conversion Nanomaterial Doped with AuNPs
Source: Int J Mol Sci. 2022 Jan 22;23(3):1227. doi: 10.3390/ijms23031227 (PMC8835744; doi:10.3390/ijms23031227)
Supplement: Supplementary file 1 [file ijms-23-01227-s001.zip › ijms-1564329-supplementary.pdf]

International Journal of Molecular Sciences

MDPI

*Supplementary Materials*

## 1. Synthesis and Characterization of Nanoparticles

### *1.1. Applied Chemicals*

$Y_2O_3$ (99.99%), $Yb_2O_3$ (99.99%), $Er_2O_3$ (99.99%), nitric acid (68%), sodium fluoride (99.99%), citric acid (99.99%), cyclohexane (99.5%), $HAuCl_4$ (99.99%), ethylenediaminetetraacetic acid (EDTA, ≥99%), sodium hydroxide (≥98%) and polyethylene pyrrolidone (PVP, average molecular weight of 1,000,000–1,500,000) were purchased from Aladdin. The cell counting kit 8 (CCK-8) assay kit was purchased from BOVOGEN. All chemicals were used as-received without additional purification.

### *1.2. Synthesis of Au Nanoparticles*

By dropping, 60 mL of 0.05 mol/L of citric acid solution was added to 3 mL of 0.02 mol/L of $HAuCl_4$ to obtain a mixture. Moreover, after 5 min of continuous stirring, the solution was transferred to a 100 mL reactor and placed in an oven for the reaction at 180 °C for 12 h. The reaction was then cooled to room temperature, washed, and centrifuged to obtain solid Au nanoparticles, and then added to 10 mL of deionized water and PVP, and then placed in a test tube to prepare the sol for use.

### *1.3. Synthesis of Au-UCNPs*

$RE_2O_3$ (RE = Y, Yb, Er) was heated to achieve complete dissolution in excess nitric acid and then transferred to a vacuum system for evaporation to obtain a solid $RE(NO_3)_3$, which was then dissolved in deionized water and recrystallized twice. A certain amount of solid $RE(NO_3)_3$ was dissolved in deionized water, and EDTA (molar ratio of EDTA: $RE(NO_3)_3$ = 1:1) was added and stirred at 600 rpm for 1 h, the mixture was then weighed and dissolved in sodium fluoride in deionized water by ultrasound, and then the solution was added and stirred at 600 rpm for 1 h. Finally, the pH value was adjusted to 5.5 with NaOH and add 10 ml of gold solution, and then place it in a hydrothermal kettle to react at 190 °C for 24 h. The reaction products were cooled, centrifuged, and washed twice with ethanol/deionized water (1:1 v/v), and dried in vacuum at 80 °C for 3 h. The resultant powder is dispersed in cyclohexane for later use.

### *1.4. DSPE-PEG$_{2K}$ Modified Au-UCNPs*

First, 6 mL of Au-UCNPs (0.4 mmol) dispersed in chloroform were mixed with 20 mL of DSPE-PEG$_{2K}$ (100 mg) chloroform solution in 5 mL open glass bottles. After heating at 75°C for 5 min to remove the chloroform, adding 24 mL water to complete the ultrasonic dispersion, stirring at 75 °C for 10 min, cool to room temperature, centrifuged at 18,000 rpm for 8 min to take the precipitate, and adding 1 mL of normal saline to disperse, large particles were removed by centrifugation at 5000 rpm for 5 min and then dried by a blast at 75 °C.

### *1.5. Characterization*

Transmission electron microscopy (TEM) measurements were performed on a JEOL 2011 microscope operating at 200 kV. All samples were first dispersed in ethanol and then collected using a Cu grid covered with a carbon film for measurement. To determine the elemental composition of the samples, energy-dispersive X-ray spectroscopy (EDS) of the samples was performed on a JEOL 2010 EDS instrument using high-resolution transmission electron microscopy (HRTEM) measurements. Inductively coupled plasma-atomic emission spectrometry (ICPAES) was performed using a Perkin Elmer 7300DV apparatus. Scanning electron microscopy (SEM) images were obtained using a Philips XL30 electron microscope operating at 20 kV. Before this characterization, an Au film was sprayed on

the sample. The upconversion luminescence spectrum was obtained using a spectrum analyzer (ANDO AQ6317, Japan). The sample was placed in a 1.0 cm path length support, which was excited using a 980 nm CW semiconductor diode laser (Pmax 800 mW, 1000 mA). The upconversion luminescence spectrum was obtained by the spectrophotometer using a multimode fiber having a core diameter of 0.6 mm. The distance between the top of the fiber and sample is ~2 mm. A spectrum analyzer (ANDO AQ6317, Japan) was used to get the up-conversion luminescence spectra. The specimen was positioned in a 1.0 cm path length support and excited by utilizing a 980 nm CW semiconductor diode laser (Pmax 800 mW, 1000 mA). The up-conversion luminescence spectrum was acquired through the spectrophotometer having a multimode fiber with a core diameter of 0.6 mm. The top of the fiber was ~2 mm away from the specimen. Thermal imager (FOCUS 280DS) was used to characterize the photoacoustic properties of photographic materials. HORIBA laser and power density meter are used to characterize photothermal properties.

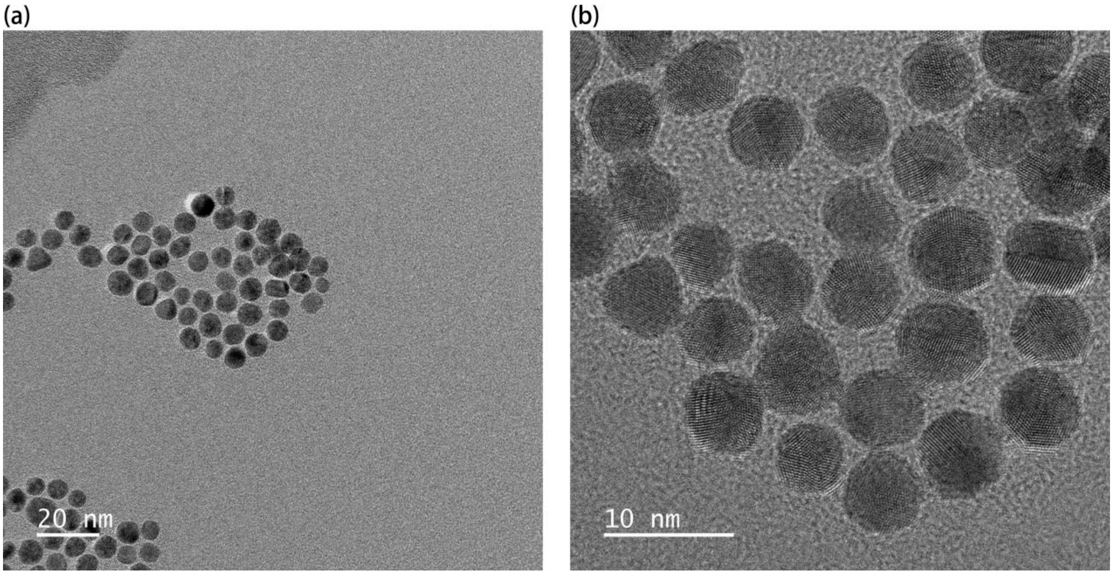


**Figure S1.** (**a**) TEM image of Au nanoparticles and (**b**) lattice of (**a**).

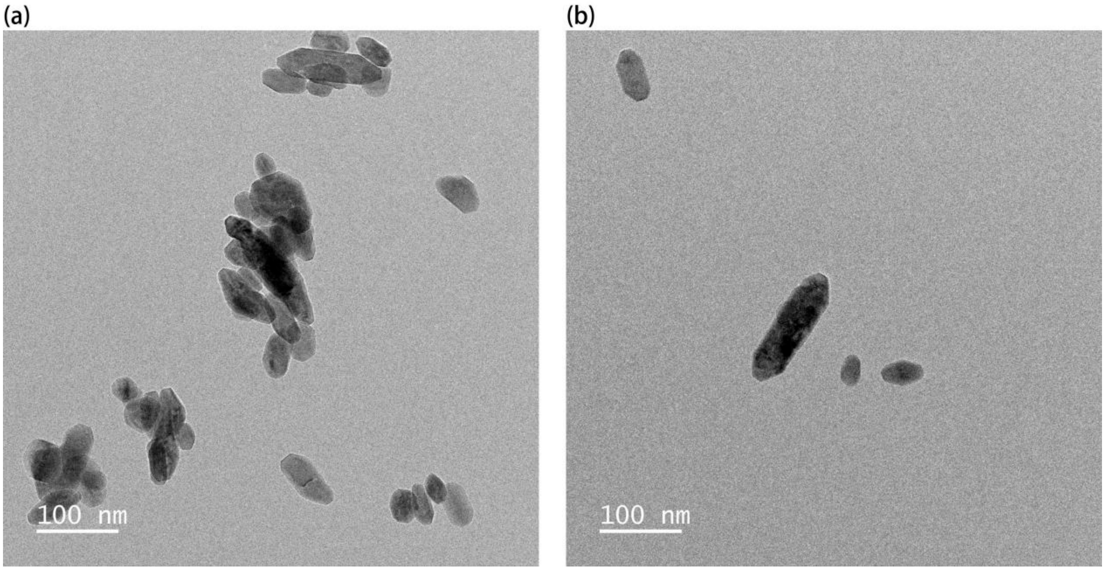


**Figure S2.** TEM image of Au-UCNPs.

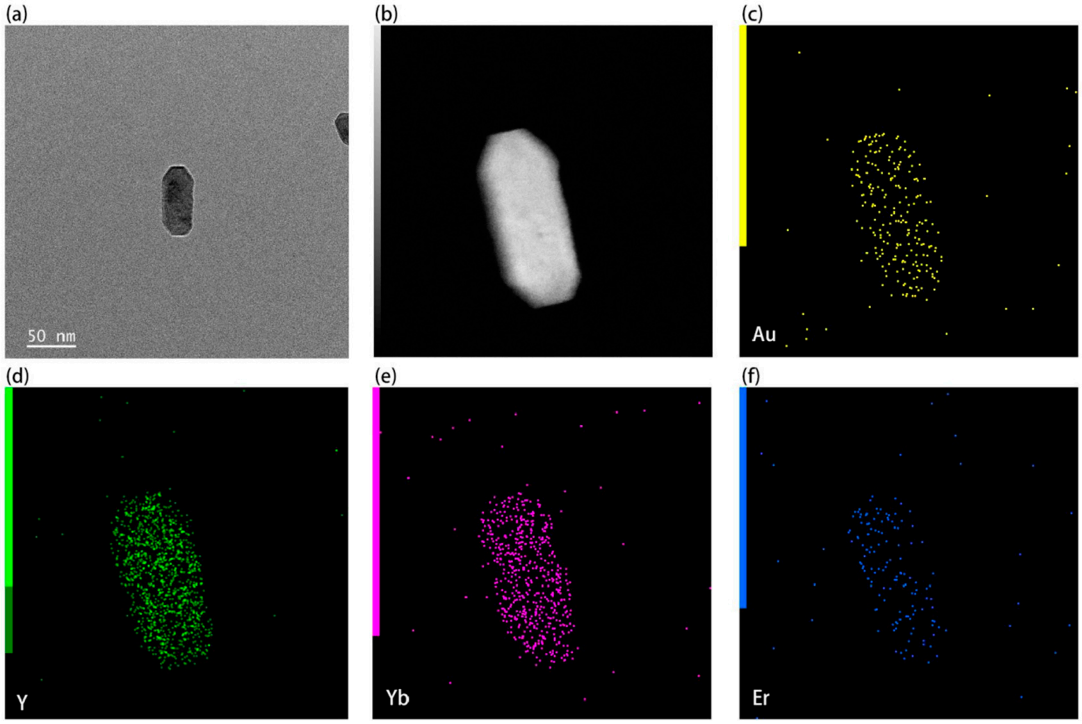


**Figure S3.** (**a**) TEM image of Au-UCNPs, (**b**) energy of (**a**), (**c**–**f**) mapping of (**b**).

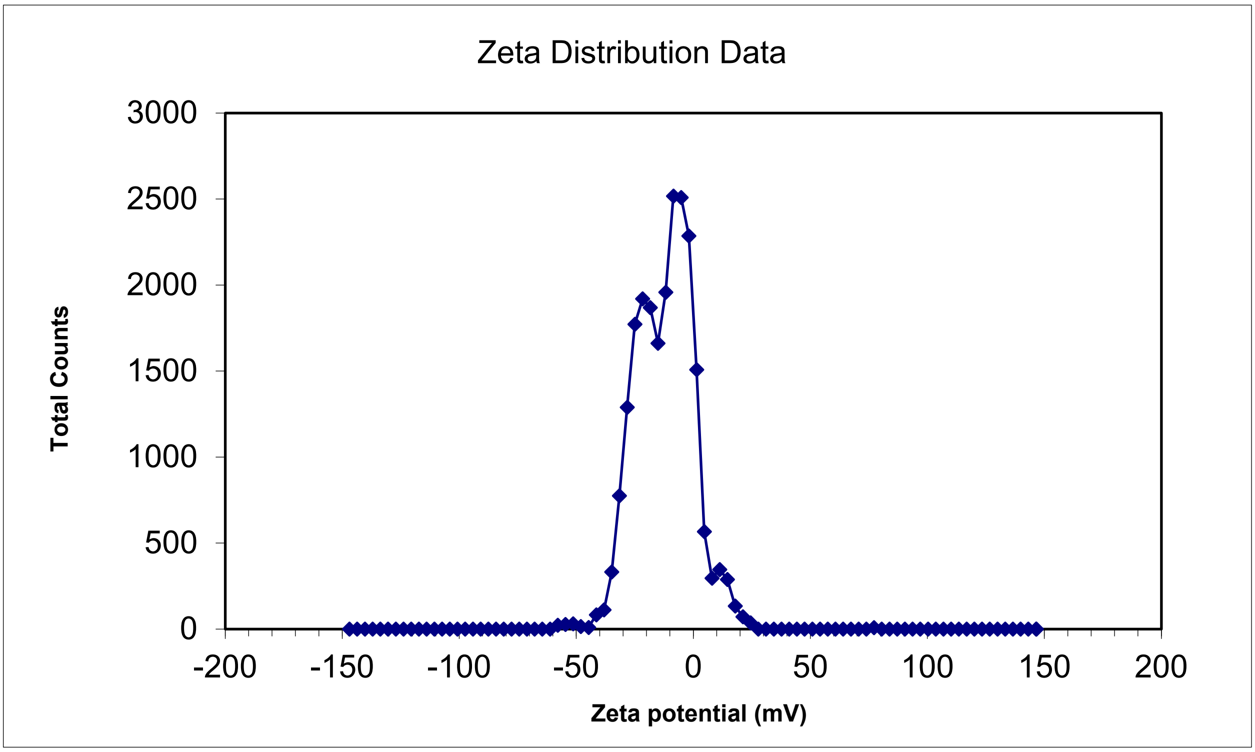


**Figure S4.** Zeta potential of gold nanoparticles.

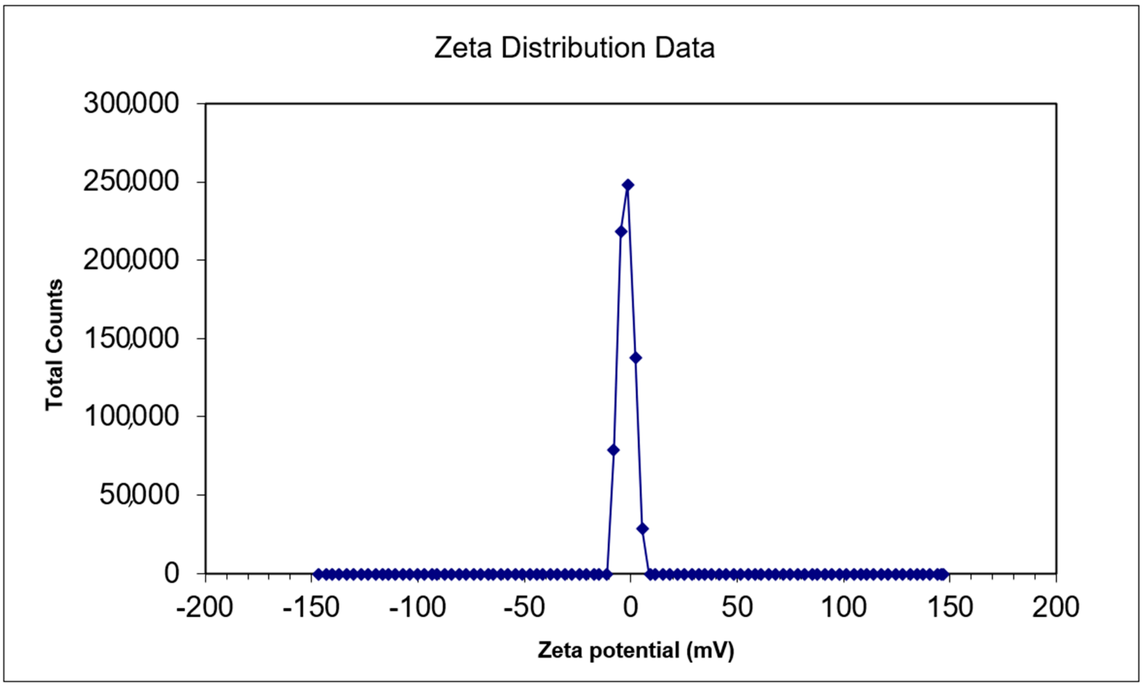


**Figure S5.** Zeta potential of Au-UCNPs.

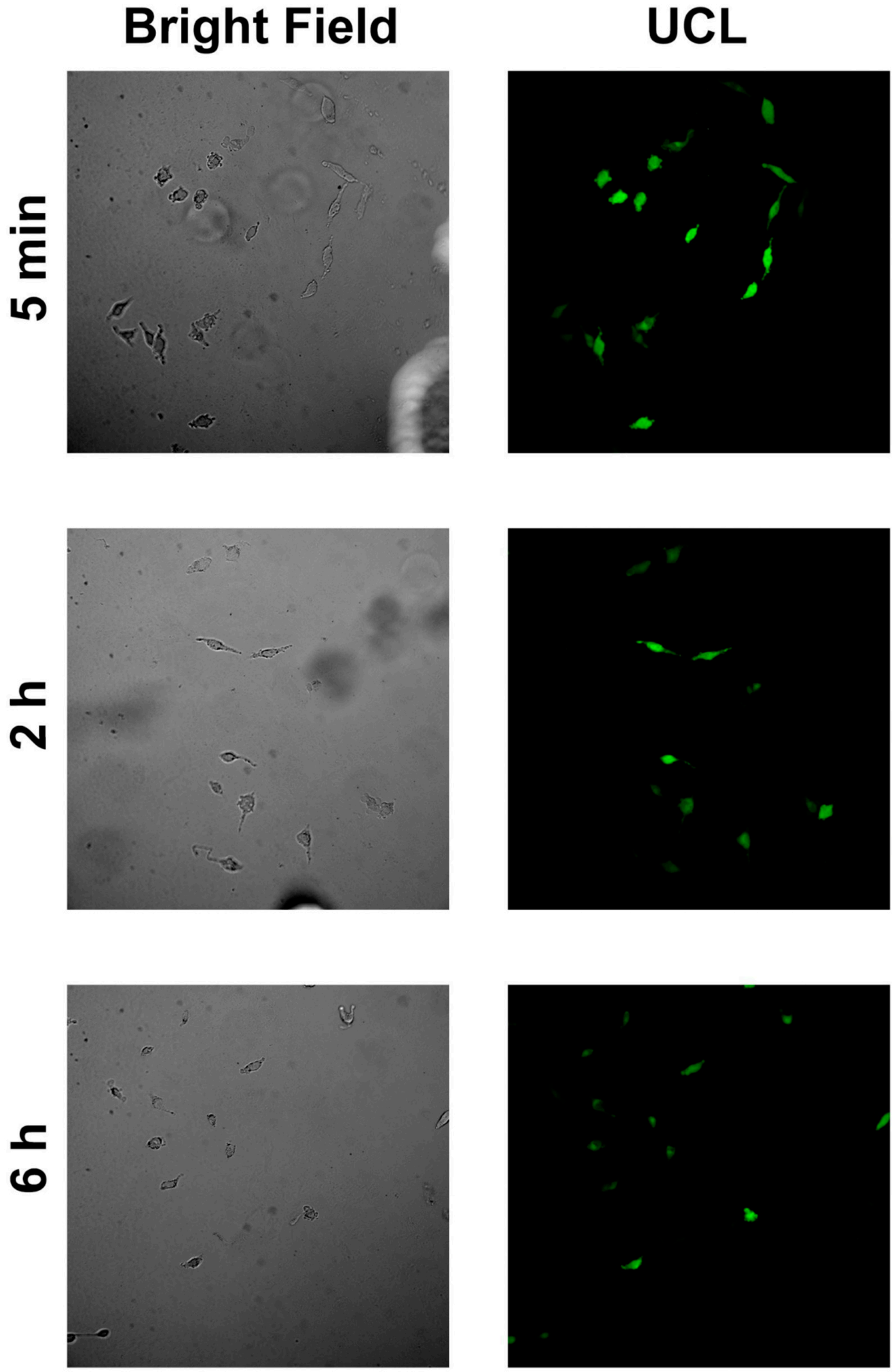


**Figure S6.** UCL images of HeLa cells incubated with Au-UCNPs-DSPE-$PEG_{2K}$ for 5 min, 2 h and 6 h.

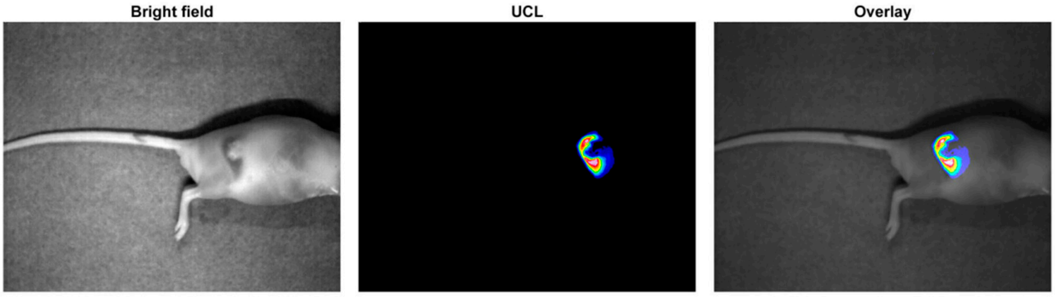


**Figure S7.** In-vivo imaging of a tumor-bearing Balb/c mouse after injection of Au-UCNPs-DSPE-$PEG_{2K}$ at the tumor site.

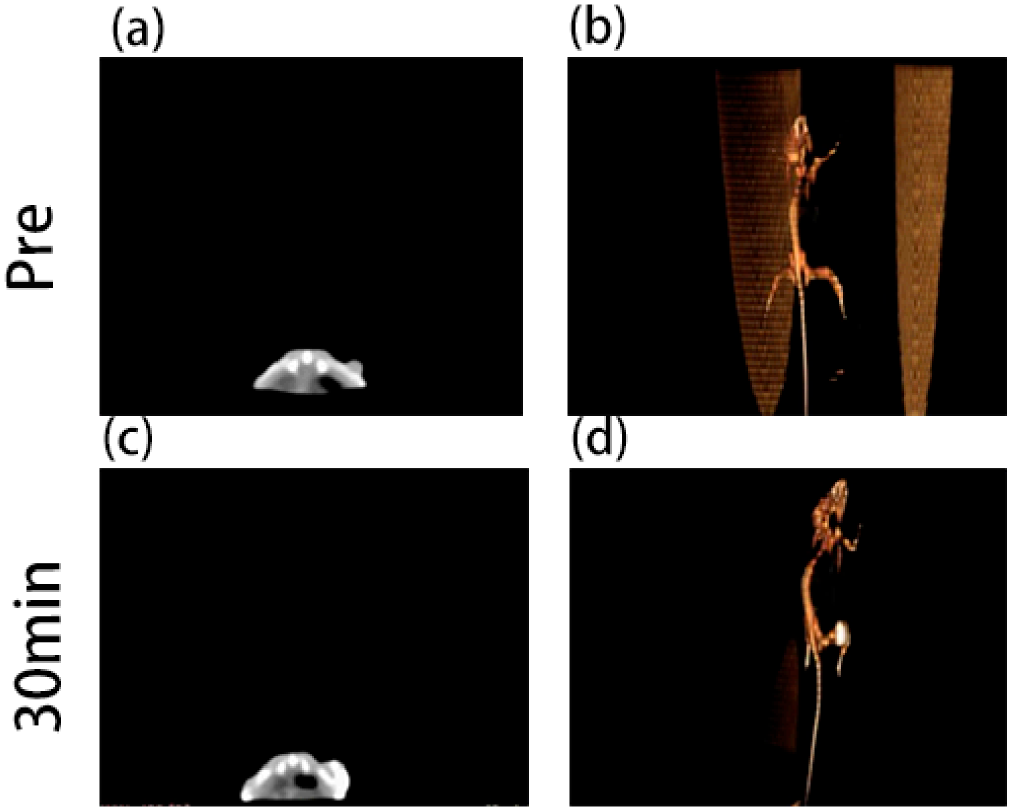


**Figure S8.** Micro-CT images before(**a**) and after(**b**) intratumor injection of Au-UCNPs-DSPE-$PEG_{2K}$ in Balb/c mice, (**c**,**d**) is the 3D model of (**a**) and (**b**) respectively.
